# Neurochemical signs of astrocytic and neuronal injury in acute COVID-19 normalizes during long-term follow-up

**DOI:** 10.1016/j.ebiom.2021.103512

**Published:** 2021-07-29

**Authors:** Nelly Kanberg, Joel Simrén, Arvid Edén, Lars-Magnus Andersson, Staffan Nilsson, Nicholas J. Ashton, Pär-Daniel Sundvall, Bengt Nellgård, Kaj Blennow, Henrik Zetterberg, Magnus Gisslén

**Affiliations:** a Department of Infectious Diseases, Institute of Biomedicine, Sahlgrenska Academy, University of Gothenburg, Gothenburg, Sweden; b Department of Infectious Diseases, Region Västra Götaland, Sahlgrenska University Hospital, Gothenburg, Sweden; c Department of Psychiatry and Neurochemistry, Institute of Neuroscience & Physiology, The Sahlgrenska Academy at the University of Gothenburg, Mölndal, Sweden; d Clinical Neurochemistry Laboratory, Sahlgrenska University Hospital, Mölndal, Sweden; e Department of Laboratory Medicine, Institute of Biomedicine, Sahlgrenska Academy, University of Gothenburg, Gothenburg, Sweden; f Wallenberg Centre for Molecular and Translational Medicine, University of Gothenburg, Gothenburg, Sweden; g King's College London, Institute of Psychiatry, Psychology and Neuroscience, Maurice Wohl Institute Clinical Neuroscience Institute, London, UK; h NIHR Biomedical Research Centre for Mental Health and Biomedical Research Unit for Dementia at South London and Maudsley NHS Foundation, London, UK; i Research, Education, Development and Innovation, Primary Health Care, Region Västra Götaland, Sweden; j General Practice/Family Medicine, School of Public Health and Community Medicine, Institute of Medicine, Sahlgrenska Academy, University of Gothenburg, Gothenburg, Sweden; k Department of Anaesthesiology and Intensive care, Institution of Clinical Sciences, Sahlgrenska Academy, University of Gothenburg, Gothenburg, Sweden; l Department of Neurodegenerative Disease, UCL Institute of Neurology, London, UK; m UK Dementia Research Institute at UCL, London, UK

**Keywords:** SARS-CoV-2, COVID-19, CNS, NfL, GFAp, GDF-15

## Abstract

**Background:**

Neurologic manifestations are well-recognized features of coronavirus disease 2019 (COVID-19). However, the longitudinal association of biomarkers reflecting CNS impact and neurological symptoms is not known. We sought to determine whether plasma biomarkers of CNS injury were associated with neurologic sequelae after COVID-19.

**Methods:**

Patients with confirmed acute COVID-19 were studied prospectively. Neurological symptoms were recorded during the acute phase of the disease and at six months follow-up, and blood samples were collected longitudinally. Healthy age-matched individuals were included as controls. We analysed plasma concentrations of neurofilament light-chain (NfL), glial fibrillary acidic protein (GFAp), and growth differentiation factor 15 (GDF-15).

**Findings:**

One hundred patients with mild (*n* = 24), moderate (*n* = 28), and severe (*n* = 48) COVID-19 were followed for a median (IQR) of 225 (187–262) days. In the acute phase, patients with severe COVID-19 had higher concentrations of NfL than all other groups (all *p* < 0·001), and higher GFAp than controls (*p* < 0·001). GFAp was also significantly increased in moderate disease (*p* < 0·05) compared with controls. NfL (*r* = 0·53, *p* < 0·001) and GFAp (*r* = 0·39, *p* < 0·001) correlated with GDF-15 during the acute phase. After six months, NfL and GFAp concentrations had normalized, with no persisting group differences. Despite this, 50 patients reported persistent neurological symptoms, most commonly fatigue (*n* = 40), “brain-fog” (*n* = 29), and changes in cognition (*n* = 25). We found no correlation between persistent neurological symptoms and CNS injury biomarkers in the acute phase.

**Interpretation:**

The normalization of CNS injury biomarkers in all individuals, regardless of previous disease severity or persisting neurological symptoms, indicates that post COVID-19 neurological sequelae are not accompanied by ongoing CNS injury.

**Funding:**

The Swedish State Support for Clinical Research, SciLifeLab Sweden, and the Knut and Alice Wallenberg Foundation have provided funding for this project.

**Research in context**

**Evidence before this study**

We reviewed the literature using PubMed (April 8, 2021) and searched for studies on biomarkers in plasma using the search terms (“plasma” OR “serum”) AND “biomarkers” AND (“COVID-19” OR “SARS-CoV-2”) AND (neurology OR central nervous system OR neuronal OR neurological). The studies identified, including our own previous reports, found neurochemical evidence of CNS injury in individuals with severe disease, including increases in plasma and cerebrospinal fluid (CSF) concentrations of neurofilament light (NfL) and glial fibrillary acidic protein (GFAp). When adding the search terms (“follow-up” OR “longitudinal” OR “post-covid” OR “complications”), only a single small exploratory cross-sectional study was identified. No clinical studies investigating the pathophysiology behind persisting neurological symptoms following SARS-CoV-2 infection could be found. The increasing epidemiological literature describing post COVID-19 condition needs to be complemented by studies investigating possible underlying pathogenic mechanisms.

**Added value of this study**

To the best of our knowledge, this is the first study reporting longitudinal data on plasma biomarkers (NfL and GFAp) of CNS injury in relation to neurological symptoms in patients recovering from COVID-19. We found that CNS injury biomarkers increase with greater disease severity during the acute phase of COVID-19 but normalize in all patients at follow-up. Persisting self-reported neurological and cognitive symptoms were common in patients, regardless of disease severity, but were not associated with biomarker evidence of CNS injury.

**Implications of all the available evidence**

Normalization of CNS injury biomarkers in all individuals, regardless of previous disease severity or persisting neurological symptoms, indicate that post COVID-19 neurological sequelae are not accompanied by ongoing CNS injury. Although injury biomarkers commonly increase in severe acute COVID-19, further investigations into the causes of post-infectious sequelae are needed.

Alt-text: Unlabelled box

## 1 Introduction

Central nervous system (CNS) involvement has been described in coronavirus disease 2019 (COVID-19) patients since the beginning of the severe acute respiratory syndrome coronavirus 2 (SARS-CoV-2) pandemic.[1,2] Headache, fatigue, dysgeusia, and anosmia are common in both mild and severe cases of COVID-19, while signs of CNS dysfunction, (including encephalopathy) and inflammatory CNS disorders (including encephalitis) have primarily been reported in patients with severe COVID-19 [2], [3], [4], [5]. Several mechanisms have been proposed as contributing to the neuropathogenesis of SARS-CoV-2. Although conclusive evidence of active viral infection of the CNS is still lacking, other effects of SARS-CoV-2 infection, including CNS immune activation secondary to systemic inflammatory responses, microvascular injuries, thromboembolic events, or unspecific hypoxic/toxic consequences of severe disease, may all contribute to the neurological manifestations of COVID-19 [1]. Our previous findings have demonstrated evidence of astrocytic and axonal injury in the acute phase of COVID-19 by analysing glial fibrillary acidic protein (GFAp), a major structural protein in the astroglial cytoskeleton and a biomarker of astrocytic activation/injury [6], and neurofilament light chain (NfL), an intra-axonal structural protein and a biomarker of neuronal injury [7]. We concluded that changes in these CNS damage biomarkers were more pronounced in hospitalized patients compared to non-hospitalized individuals and healthy controls [8]. Results confirming this have subsequently been reported in other studies [9,10]. In addition, in the search for useful biomarkers to predict COVID-19 outcomes, cytokine growth differentiation factor 15 (GDF-15) was recently proposed as a biomarker of general disease severity and unfavourable outcomes [11].

It has become increasingly evident that after the acute phase of COVID-19 many patients suffer from persistent neurologic disability, often including lethargy, fatigue, or impaired cognitive function [12]. It has been suggested that cognitive examinations should be included in the clinical follow-up of COVID-19 patients, especially in the case of those who develop cerebrovascular and neurologic complications during the acute stage [13]. The long-term impact of COVID-19 on the CNS remains largely unknown, and thus far there have been no published studies that have investigated the long-term trajectories of biomarkers of disease severity and neurological involvement in COVID-19, nor how they relate to persistent neurological symptoms.

In our study, we have prospectively examined the dynamic changes in plasma biomarkers of CNS injury (GFAp and NfL) and persistent neurological symptoms in a cohort of patients with mild, moderate, or severe COVID-19 during the acute phase of the disease, and in subsequent follow-up in comparison with uninfected controls to determine the long-term CNS impact of COVID-19.

## 2 Methods

### 2.1 Participants and study design

We recruited patients with confirmed COVID-19 diagnosed between 21 February and 5 November 2020 at the Department of Infectious Diseases, Sahlgrenska University Hospital, Gothenburg, Sweden. The recruitment period was not predetermined. Patients below age 18 or with other pre-existing active neurological diseases were excluded. Upon enrolment, previous medical history, current physical status, symptoms, and medication list were recorded in an electronic medical database. The patient cohort was divided into three groups according to disease severity, as defined by the WHO Clinical Progression Scale [14]. Mild disease included asymptomatic or symptomatic ambulatory patients; moderate disease included hospitalized patients receiving oxygen therapy by mask or nasal cannula; and severe disease included hospitalized patients in need of high flow nasal oxygen (HFNO) or mechanical ventilator. Age-matched individuals were included as controls (healthcare workers recruited locally).

Hospitalized patients were admitted to the Department of Infectious Diseases, Sahlgrenska University Hospital. Blood samples were collected at admission and after 4 weeks for patients who remained hospitalized. Blood samples from patients with mild COVID-19 were collected during the acute phase in the outpatient clinic of the same department. Follow-up consultations were held between May 2020 and May 2021 by a registered nurse or a physician. Participants were interviewed and were also asked to complete a self-reporting symptom questionnaire and the Karolinska Exhaustion Disorder Scale (KEDS) in order to assess symptoms of stress-induced exhaustion disorder [15]. The symptom questionnaire included self-reported comorbidities, current medications, symptoms experienced during acute infection, date of symptom onset, and perceived disease severity. Patients also reported and graded any new or persistent symptoms, including fatigue, “brain-fog”, and changes in cognition (i.e., memory or concentration).

### 2.2 Ethics

The study was approved by the Swedish Ethics Review Authority (2020-01771). All participants were enrolled after giving their written informed consent.

### 2.3 Procedures

SARS-CoV-2 infection was confirmed with real-time reverse transcription-polymerase chain reaction (RT-PCR) analysis of nasal and throat swab specimens, as previously reported [16]. Nucleic acid was extracted from the clinical samples in a MagNA Pure 96 instrument using the Total Nucleic Acid isolation kit (Roche, Basel, Switzerland). RT-PCR targeting the RNA-dependent RNA polymerase (RdRP) region was performed by a QuantStudio 6 instrument (Applied Biosystems, Foster City, CA, USA) using a probe and the primers RdRP_Fi, GTCATGTGTGGCGGTTCACT and RdRP_Ri, CAACACTATTAGCATAAGCAGTTGT.

All plasma GFAp and NfL measurements were performed at the Clinical Neurochemistry Laboratory at Sahlgrenska University Hospital by board certified laboratory technicians blinded to clinical data on an HD-X Analyzer, as described by the manufacturer (Quanterix, Billerica, MA, USA) [17,18]. Plasma GFAp and NfL were measured using commercially available GFAp discovery (#502656) and Nf-Light (#502296) kits (Quanterix, Billerica, MA, USA). GDF-15 was measured using an Elecsys electrochemiluminescence immunoassay on the Cobas platform (Roche Diagnostics, Rotkreuz, Switzerland). A single batch of reagents was used; inter- and intra-assay coefficients of variation were below 15% for all analytes.

C-reactive protein (CRP) (mg/L) and lymphocyte count (x 10^9^/L) were analysed at the Sahlgrenska University Hospital Clinical Chemistry Laboratory. The highest CRP concentration and the lowest lymphocyte count of each patient during the acute phase of the disease were recorded.

### 2.4 Statistical analyses

Graphs and corresponding statistical analyses were generated using Prism (GraphPad Software version 8·00, La Jolla, CA, USA), whereas all other analyses were performed in SPSS statistics (IBM SPSS version 27). Plasma biomarkers were log_10_ transformed to reduce skewness. All data are reported as median and interquartile range (IQR), unless otherwise indicated. Associations between biomarkers were performed using Pearson correlation. Group differences of plasma biomarkers were estimated by analysis of covariance (ANCOVA), using age as covariate. Paired t-tests were used to assess biomarker changes between timepoints. Binary logistic regressions were performed to investigate if the presence of neurological symptoms at follow-up were affected by the interval between the acute phase and time to follow-up or by higher concentration of biomarkers in the acute phase. All tests were two-tailed; a *p*-value < 0·05 was considered statistically significant.

### 2.5 Role of the funding source

Those who funded the study had no role in the study design, data collection, data analysis, data interpretation, or writing of the report.

## 3 Results

We recruited 100 patients with confirmed COVID-19 for this study. Findings from the acute phase of the disease have previously been reported for 30 of the included individuals [8]. The median (IQR) age in the COVID-19 cohort was 55 (48–65) years; 57 (57%) were male (Table 1). In the group of patients with mild disease (*n* = 24), 14 (58%) were female and had a significantly lower median (IQR) BMI of 25 (22–28) than the other groups (*p* < 0·001). Of those patients with moderate (*n* = 28) and severe (*n* = 48) disease, 20 (71%) and 38 (79%), respectively, were male and had significantly more comorbidities compared to individuals with mild disease. Hypertension and overweight/obesity were the most common underlying conditions; 12 (43%) patients had hypertension and 27 (96%) patients were overweight/obese in the group of moderately ill individuals. Twenty-four (50%) patients had hypertension, and 33 (63%) were overweight/obese in the severely ill group. Furthermore, in the severe disease group, 15 (31%) patients were on HFNO, and 33 (69%) patients required mechanical ventilation. Two patients had a past history of stroke, one in 2018 and the other in 2009. Thirteen patients were receiving ongoing treatment with metformin and 27 were treated with angiotensin-converting enzyme inhibitors (ACEIs)/angiotensin receptor blockers (ARBs). In addition, 51 age-matched control subjects were included in this study. The median (IQR) age in the control group was 55 (44–63) years; 38 (75%) were female, 7 (14%) had hypertension, and 3 (6%) had diabetes.

**Table 1:** Patient characteristics.

	*All participants (n = 151)*					
	*Controls* (*n* = 51)	*Mild* (*n* = 24)	*Moderate* (*n* = 28)		*Severe* (*n* = 48)	*p-value*
*Demographic characteristics*						
Age, median (IQR), years	55 (44–63)	55 (41–60)	55 (52–64)	58 (50–67)		·308
*Sex, n (%)*						
Female	38 (75)	14 (58)	8 (29)	10 (21)		< ·001
Male	13 (25)	10 (42)	20 (71)	38 (79)		
*Comorbidities, n (%)*						
Hypertension	7 (14)	2 (8)	12 (43)	24 (50)		< ·001
Overweight/Obesity	..	9 (37)	27 (96)	33 (63)		< ·001
BMI median (IQR) kg/m^2^	..	25 (22–28)^a^	31 (29–34)^a^†	29 (25–32)^a^		< ·001
Diabetes	..	1 (4)	7 (25)	8 (17)		·045
Coronary heart disease	3 (6)	1 (4)	1 (4)	4 (8)		·638
*Blood samples median (IQR)*						
CRP, mg/L	..	6·0 (2·5–9·5) b	92 (48–130)†	240 (170–320)†‡		< ·001
Lymphocyte count 10^9^/L	..	1·2 (1–2) b	1·0 (0·8–1·3)	0·7 (0·6–1·0)†		·001
GFAp, pg/mL	129 (104–172)	135 (107–223)	178 (123–249)†*	232(161–340)*†		< ·001
NfL, pg/mL	10 (7·2–15)	8·7 (6·1–16)	11 (6·2–16)	21(10–32)*†‡		< ·001
GDF-15, pg/mL	703 (501–949)	748 (586–1·087)	3450 (2·337–4·105)*†	3562 (2·458–5·880)*†		< ·001

a BMI calculated in 19 patients with mild, 27 patients with moderate, and 33 patients with severe disease

b CRP and lymphocyte count available for 8 patients with mild disease * = significantly different from controls; † = significantly different from mild cases, *p* < 0·05 (ANCOVA); ‡ = significantly different from moderate cases, *p* < 0·05 (ANCOVA) Group comparisons were made using univariate linear models test with post-hoc least significant difference test for continuous variables, and Fisher's exact test for dichotomous variables. Abbreviations: GDF-15, growth differentiation factor 15; NfL, neurofilament light; GFAp, glial fibrillary acidic protein; BMI, body mass index; CRP, C-reactive protein; N/S, non-significant

At baseline, the most frequent neurological symptoms were headache (41%) and dysgeusia (11%). Myalgia, hyposmia, and dysgeusia were more common in patients with mild, as opposed to severe disease, whereas altered cognition (memory or concentration) was most common in patients with severe disease (Table S1).

At follow-up, self-reported symptom questionnaires were completed by 97 patients at a median (IQR) of 225 (187–262) days after onset of symptoms: 267 (260–278) days for mild; 147 (105–224) days for moderate; and 220 (192–253) days for severe disease. In total 50 patients (50%) reported one or more neurological symptoms: 11 (11%) for mild, 14 (14%) for moderate, and 25 (25%) for severe disease. The most common symptoms reported at follow-up were fatigue (41%), “brain-fog” (30%), and cognitive impairment such as memory loss and lack of concentration (26%) (Table 2). However, no significant differences in the frequency of any symptoms were found among the groups. Binary logistic regression models showed no persisting neurological symptoms that were significantly associated with time to follow-up consultation in months (Table S2).

**Table 2:** Neurological symptoms at follow-up *n* (%).

	Mild (*n* = 24)	Moderate (*n* = 26)	Severe (*n* = 47)	*p*-value*
Any	7 (29)	13 (46)	26 (54)	·75
Fatigue	9 (38)	11 (42)	20 (42)	·59
“Brain-fog”	6 (25)	10 (38)	13 (27)	·92
Changes in cognition	4 (17)	6 (23)	15 (31)	·34
Hyposmia	2 (8)	1 (4)	1 (2)	..
Dysgeusia	2 (8)	2 (7)	1 (2)	..

*Fisher's exact test was used to explore group differences in the prevalence of neurological symptoms at follow-up

Baseline blood samples were collected at a median (IQR) of 10 (7–13) days after symptom onset: 11 (3–17) days for mild, 10 (8–13) days for moderate, and 10 (7–13) days for severe disease. Participants had blood samples collected at a median (IQR) of 3 (3–3) time points and were followed for a median (IQR) of 225 (187–262) days.

**Fig. 1:**
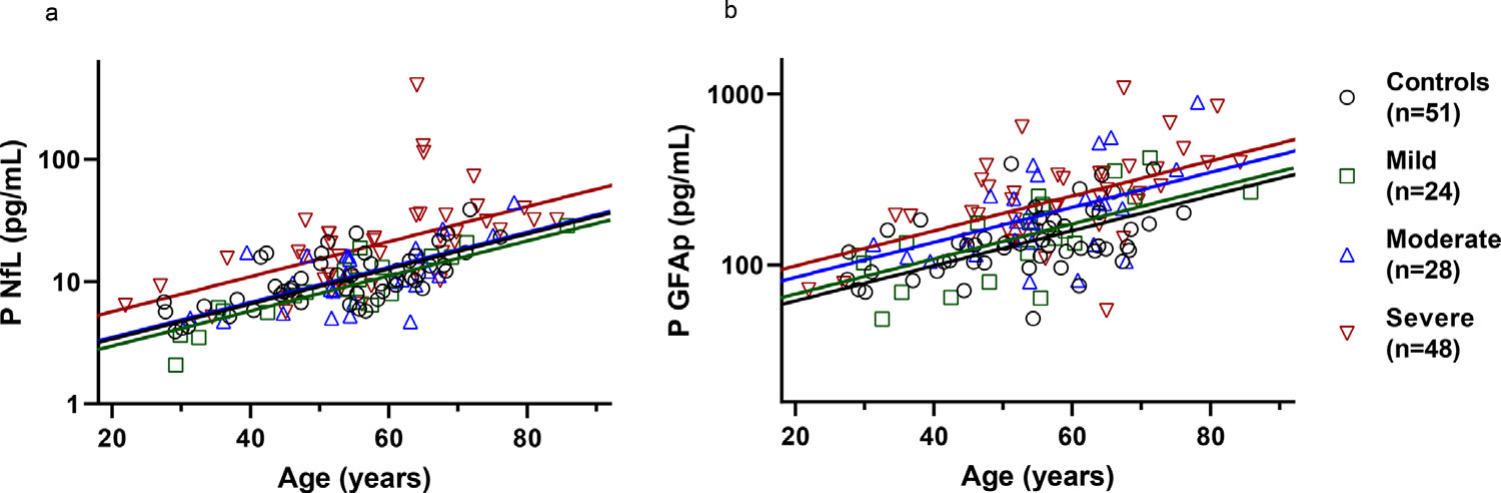
NfL and GFAp during acute phase of disease. Plasma concentrations of NfL (a) and GFAp (b) shown in acute phase in patients with mild, moderate, and severe disease, and controls (age on x-axis). Patients with severe disease show higher concentrations of plasma NfL than other groups (*p* < 0·001) and significantly higher GFAp concentrations compared to controls (*p* < 0·001, ANCOVA). GFAp also higher in patients with moderate disease compared to controls (*p* = 0·028, ANCOVA). All *p*-values are adjusted for age. *Abbreviations*: NfL, neurofilament light; GFAp, glial fibrillary acidic protein.

**Fig. 2:**
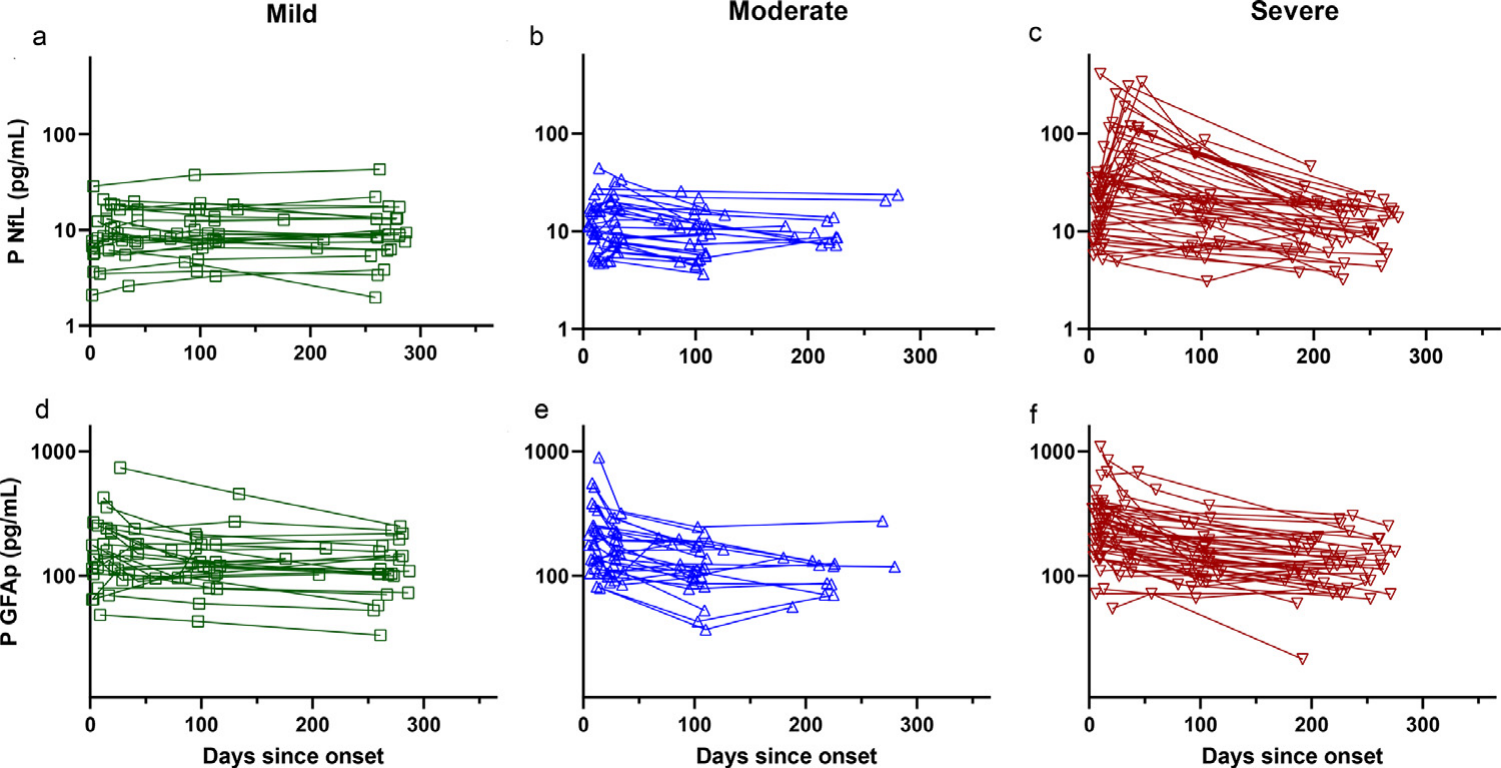
Longitudinal trajectories of biomarkers reflecting CNS pathophysiology Longitudinal trajectories of patients displayed by diagnostic category. NfL concentrations shown for patients with mild (a), moderate (b), and severe (c) disease. GFAp concentrations for same groups also shown (d–f). Concentrations of NfL increased in severely ill patients in blood samples taken at 30–70 days after onset (*p* < 0•001, t-test), whereas GFAp decreased in both moderately and severely ill patients (*p* < 0•001, t-test). *Abbreviations*: NfL, neurofilament light; GFAp, glial fibrillary acidic protein.

In the acute phase of COVID-19 (< 21 days after symptom onset; *n* = 92), NfL and GFAp were significantly correlated with age (NfL: *r* = 0·63, GFAp: *r* = 0·55), and with each other (*r* = 0·48) in the whole sample (all *p* < 0·001, Pearson correlation) (Fig. S1). Patients with severe disease had higher concentrations of plasma NfL than all other groups (*p* < 0·001, ANCOVA) and significantly higher GFAp concentrations compared to controls (*p* < 0·001, ANCOVA) (Fig. 1). GFAp was also higher in patients with moderate disease compared to controls (*p* = 0·028, ANCOVA). In addition, plasma NfL concentrations in severely ill patients continued to increase in blood samples taken 30–70 days after symptom onset, as compared to acute phase sampling (*n* = 18) (*p* < 0·001, t-test), whereas GFAp decreased both among patients with severe (*p* < 0·05, t-test) and moderate (*p* < 0·001, t-test) disease (Fig. 2). However, no group differences were found in plasma NfL concentrations at follow-up, regardless of initial COVID-19 severity (Fig. S2). Similarly, no significant differences in GFAp concentrations persisted between groups at follow-up (Fig. S2). When assessing the impact of the degree of CNS injury during the acute phase using binary logistic regression (reflected by NfL and GFAp concentrations), we found that higher biomarker concentrations were not related to any of the symptoms reported at follow-up (Table S3).

**Fig. 3:**
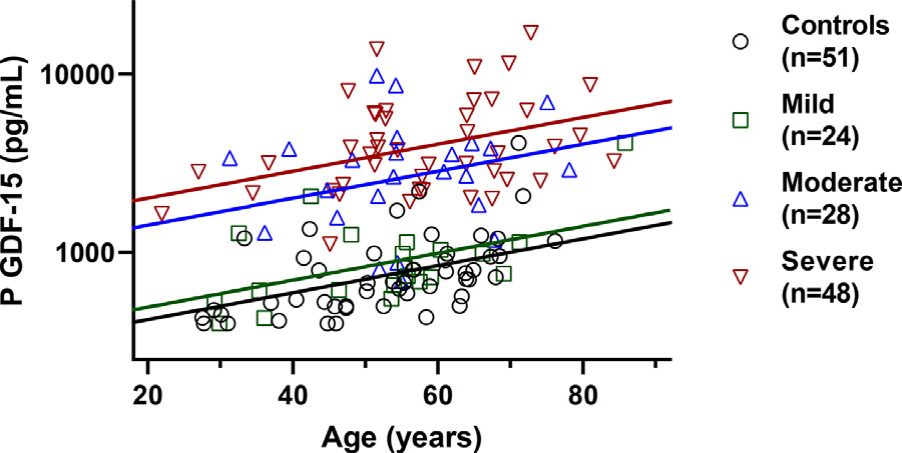
Biomarker evidence of systemic impact during acute phase of COVID-19. In acute phase of disease, patients with severe and moderate disease had higher plasma GDF-15 concentrations compared to others with mild disease and controls (*p* < 0·001, ANCOVA). Age of patients shown on x-axis. *P*-values are adjusted for age. *Abbreviations*: GDF-15, growth differentiation factor 15.

In addition to acute phase concentrations in lymphocyte count and CRP, we longitudinally evaluated GDF-15 as a marker of disease severity to investigate its relation to CNS impact. During the acute phase of COVID-19, patients with severe and moderate disease had markedly higher GDF-15 concentrations compared to individuals with mild disease and to controls (*p* < 0·001, ANCOVA) (Fig. 3). Similarly, CRP was higher in patients with severe COVID-19 compared to all other groups (*p* < 0·001, ANCOVA), while lymphocyte counts were lower in severe, as opposed to mild disease (*p* < 0·01, ANCOVA) (Table 1). CRP was positively associated with GDF-15 (*r* = 0·56, *p* < 0·001, Pearson correlation), NfL (*r* = 0·31, *p* < 0·01, Pearson correlation), and negatively associated with lymphocyte count (*r* = 0·26, *p* < 0·05, Pearson correlation). In addition, lymphocyte count was also negatively correlated with GDF-15 (*r* = –0·26, *p* < 0·05, Pearson correlation), as well as GFAp (*r* = –0·28, *p* < 0·05, Pearson correlation). At six months post-infection, GDF-15 concentration remained significantly higher in the severe and moderate COVID-19 groups, compared to patients with mild COVID-19 and controls (*p* < 0·05, ANCOVA) (Fig. S2).

## 4 Discussion

In this longitudinal study, we used blood biomarkers of neuroaxonal injury, astroglial activation, and disease severity to investigate the impact of SARS-CoV-2 infection on the CNS up to six months post-acute COVID-19. Despite normalization of plasma concentrations of CNS injury biomarkers, a significant number of patients still experienced persistent neurological and cognitive symptoms. However, we found no association between higher concentrations of CNS injury biomarkers during the acute phase and post-infectious neurological symptoms at a follow-up visit after approximately six months.

In the acute phase, concentrations of NfL and GFAp were increased in parallel with COVID-19 disease severity. Moreover, the CNS injury biomarkers demonstrated distinct temporal patterns over time in the different disease severity categories (Fig. 2). We have previously shown that blood concentrations of NfL and GFAp increase with disease severity during the acute phase of COVID-19 [8]. In the present study we have extended our findings to show that patients with severe illness have a persistent increase in plasma NfL at 30–70 days after symptom onset compared to patients with mild and moderate disease. In blood samples taken six months post-infection, however, NfL concentrations were no different from controls in any of the three disease severity groups. By contrast, concentrations of GFAp showed a more rapid dynamic: increased concentrations were only seen during the acute phase in patients with severe and moderate disease compared to controls. Moreover, there were no longer any group differences in GFAp concentrations at post-infection follow-up.

Besides the higher CRP and lower lymphocyte count levels, GDF-15 concentrations were higher in patients with moderate to severe COVID-19 during acute infection as compared to those with mild COVID-19 and controls, and GDF-15 concentrations remained elevated at follow-up. The increased GDF-15 concentrations seen in patients with moderate and severe disease supports the findings of a recent study where higher GDF-15 levels were associated with viremia, hypoxemia, or worse clinical outcomes in patients hospitalized with COVID-19 [11]. In contrast to what has been reported previously, metformin usage was not associated with an increase in GDF-15 levels [19].

Several mechanisms likely contribute to CNS neuropathogenesis during acute COVID-19. These include direct effects of viral infection, primarily in the olfactory mucosa; indirect effects of the systemic inflammatory response leading to activation of CNS-resident immune cells; microvascular injury and thrombosis related to the hypercoagulable state, as well as endotheliitis resulting from viral interaction with perivascular cells; and other unspecific hypoxic/toxic consequences of severe disease leading to CNS immune activation [1].

The results of our study indicate that astrocytic injury or activation occurs early in the acute phase of COVID-19, while the slower kinetic of NfL suggests a delayed neuronal injury. The early increase in GDF-15 in addition to well-characterized markers of disease severity (CRP, lymphocyte count) indicates that the magnitude of immune activation is associated with CNS injury. Intrathecal immune activation and neuroaxonal injury has also been described in studies of cerebrospinal fluid (CSF) biomarkers, where glial activation and cytokine release is a characteristic finding, suggesting an interaction between the systemic and CNS immune responses, possibly via the neurovascular interphase.

Common features of neurotropic infection (detection of viral RNA, pleocytosis, blood-brain barrier injury, increased and intrathecal immunoglobulin production) are often mild or absent in CSF studies, suggesting that direct viral CNS infection may not be the primary driver of neurological manifestations in patients with COVID-19 in the vast majority of cases [20], [21], [22], [23].

The decrease of GFAp and GDF-15 concentrations in plasma after 30–70 days indicates a gradual resolution of the acute inflammatory process, with a reduction in the levels of circulating cytokines and diminishing astrogliosis. Conversely, the sustained increase of NfL seen in patients with severe COVID-19 may reflect a delayed response to the acute phase, possibly due to Wallerian degeneration with anterograde degeneration of axons and their accompanying myelin sheath following earlier injury to the proximal portion of the neuronal cell body [24].

The long-term sequelae to SARS-CoV-2 infection is a novel, poorly researched aspect of COVID-19. Epidemiological studies have characterized what is now termed the post COVID-19 condition [25]. However, data is still very limited regarding potential pathogenetic mechanisms underlying the reported post COVID-19 condition and whether demographic factors, cultural aspects, or differences in healthcare systems might influence its characteristics or prevalence. We believe that the findings of our study may be generalized to countries with an equivalent health care system and pandemic situation but studies from other settings are needed to investigate the global generalizability of our findings. Nonetheless, studies such as ours and other investigations will be of great importance in trying to differentiate between active pathological processes and consequences of previous injuries that result in post-infectious sequelae in patients who exhibit long-term neurological or cognitive symptoms during recovery [26].

In a recent registry study, the frequency of neurological manifestations was seen to increase with disease severity [27]. Individuals were also more likely to receive a diagnosis of dementia after COVID-19, as compared to after influenza or other respiratory infections [27]. Although our study was not designed to evaluate or diagnose the presence of dementia, the data presented here suggests that the cognitive symptoms experienced by some of our participants were not accompanied by signs of persistent neurodegeneration that is an ominous feature of dementia syndromes, whereas the ability of plasma NfL to detect such alterations is supported by a vast literature [28].

Our study has some limitations. First, the timing of the blood samples collected varied because of loss to follow-up or lack of compliance with scheduled appointments. This is evident in the difference between the follow-up days after onset of symptoms in the mild and moderate/severe groups. Outpatient follow-up in the mild disease group may have been delayed due to the fact that this group could have been more capable of rescheduling missed appointments. Additionally, any neurological and cognitive symptoms present during the acute phase of the disease were documented in the patient's electronic medical records by different attending physicians. This could have affected the structure and specificity of the documentation of neurological and cognitive symptoms, which may in turn have led to under- or over-representation of the severity of symptoms. At follow-up, symptoms were self-reported and not confirmed by standardized cognitive tests and fatigue scales, thereby not allowing the consistent grading of the severity of neurological symptoms. Consequently, we could not assess a possible association between severity of neurological symptoms and biomarker concentrations. Due to infection control measures present at the time of the study, further neuroimaging and EEG studies were limited and generally not available for this report. This raises the question of underestimating the burden of neurology morbidity [29], although the presence or absence of ongoing neuronal injury may be accurately assessed with NfL [30]. In addition, the wasting of peripheral nerves, a recognized feature in ICU patients, may contribute to increased NfL concentrations, although it cannot explain an increase in GFAp [31], [32], [33].

Despite their limitations, our findings have important implications. The normalization of CNS injury biomarkers in all individuals, regardless of previous disease severity or persistent neurological symptoms, suggests that common post COVID-19 neurological sequelae are not due to active neurodegeneration or astroglial activation. However, considering the rapidly increasing number of individuals who do suffer from post-infectious neurological sequelae while recovering from COVID-19, further studies of the underlying causes and tenacious pathological processes that SARS-CoV-2 infection may initiate are urgently needed.

## Data sharing statement

Researchers who wish to apply for access to anonymized data from the present study are welcome to contact the corresponding author. Requests should be accompanied by well-defined research questions corresponding to the overall research agenda for the cohort.

## Declaration of Competing Interest

KB has served as a consultant, on advisory boards, or on data monitoring committees for Abcam, Axon, Biogen, Julius Clinical, Lilly, MagQu, Merck, Novartis, Roche Diagnostics, and Siemens Healthineers, and is a co-founder of Brain Biomarker Solutions (BBS) in Gothenburg, Sweden, a part of the GU Ventures Incubator Program (externally submitted work). HZ has served on scientific advisory boards for Denali, Roche Diagnostics, Wave, Samumed, Siemens Healthineers, Pinteon Therapeutics; and CogRx; has lectured at symposia sponsored by Fujirebio, Alzecure, and Biogen; and is a co-founder of Brain Biomarker Solutions (BBS) in Gothenburg, Sweden, part of the GU Ventures Incubator Program (external submitted work). MG has received research grants from Gilead Sciences and Janssen-Cilag, and honoraria from Amgen, Biogen, Bristol-Myers Squibb, Gilead Sciences, GlaxoSmithKline/ViiV, Janssen-Cilag, MSD, Novocure, and Novo Nordic as a speaker or scientific advisor. All other authors report no competing interests.

## Appendix Supplementary materials

Image, application 1

Image, application 2

Image, application 3

Image, application 4
