# Supplementary material for: Neurochemical signs of astrocytic and neuronal injury in acute COVID-19 normalizes during long-term follow-up
Source: eBioMedicine. 2021 Jul 29;70:103512. doi: 10.1016/j.ebiom.2021.103512 (PMC8320425; doi:10.1016/j.ebiom.2021.103512)
Supplement: Supplementary file 1 [file mmc1.docx]

Neurochemical signs of astrocytic and neuronal injury in acute COVID-19 normalizes during long-term follow-up

**Supplementary figures……………………………………………………………………………………………………...**

Figure S1. Correlations between NfL, GDF-15 and GFAp in the acute phase

Figure S2. Plasma NfL and GFAp concentrations at follow-up >175 days

**Supplementary tables……………………………………………………………………………………………………...**

Table S1. Neurological symptoms reported at baseline in COVID-19 patients

Table S2. Logistic regression models assessing the effect on time to follow up on persisting neurological symptoms

Table S3. Logistic regression models assessing the effects of plasma biomarker concentrations on neurological symptoms
