# Supplementary figures and images for: Neurochemical signs of astrocytic and neuronal injury in acute COVID-19 normalizes during long-term follow-up
Source: eBioMedicine. 2021 Jul 29;70:103512. doi: 10.1016/j.ebiom.2021.103512 (PMC8320425; doi:10.1016/j.ebiom.2021.103512)

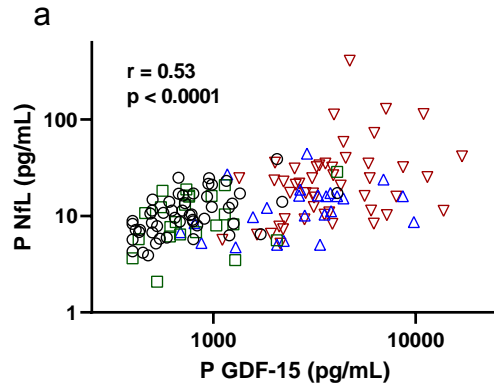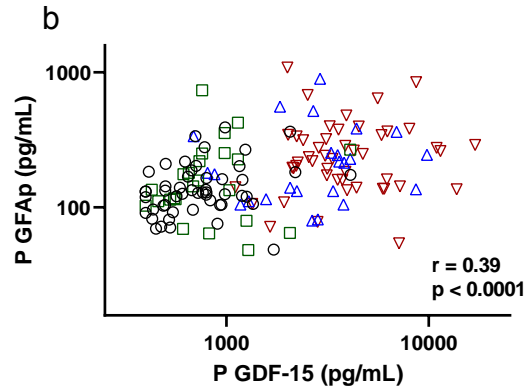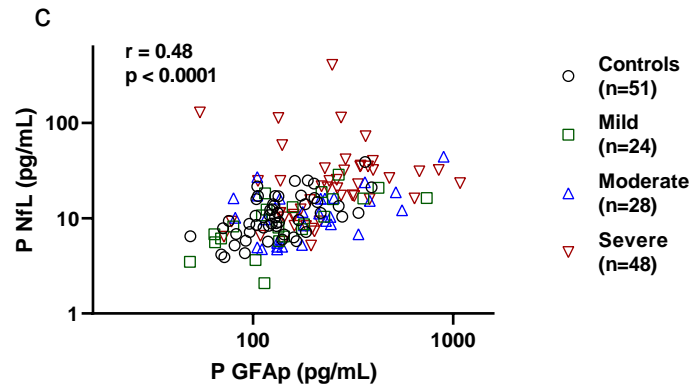

Supplement: Supplementary file 2 [file mmc2.pdf]

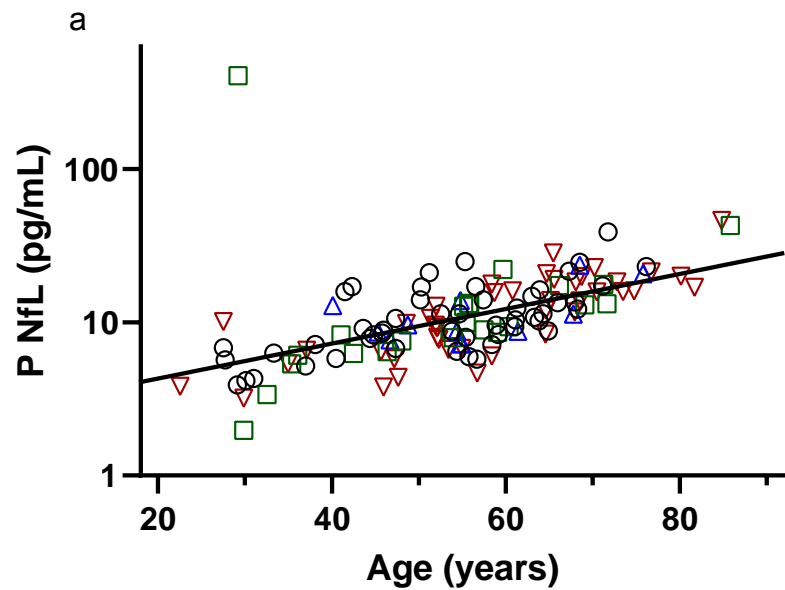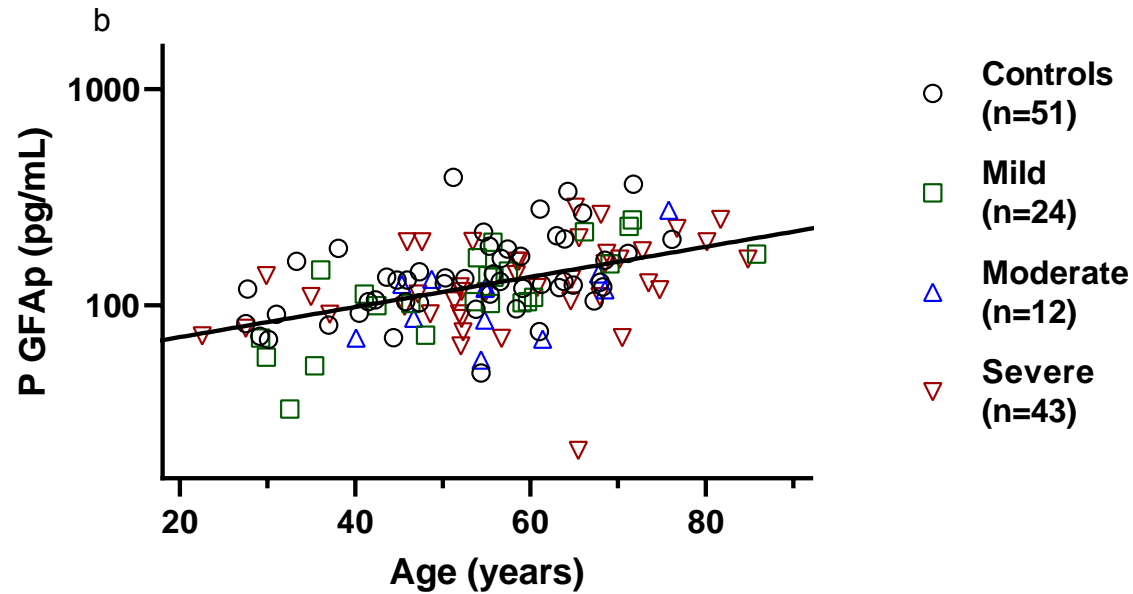

Supplement: Supplementary file 3 [file mmc3.pdf]
