# Supplementary material for: Neurochemical signs of astrocytic and neuronal injury in acute COVID-19 normalizes during long-term follow-up
Source: eBioMedicine. 2021 Jul 29;70:103512. doi: 10.1016/j.ebiom.2021.103512 (PMC8320425; doi:10.1016/j.ebiom.2021.103512)
Supplement: Supplementary file 4 [file mmc4.docx]

**Supplementary appendix**

Neurochemical signs of astrocytic and neuronal injury in acute COVID-19 normalizes during long-term follow-up

**Table of contents**

**Authors……………………………………………………………………………………………………**

**Supplementary figures……………………………………………………………………………………………………...**

Figure S1. Correlations between NfL, GDF-15 and GFAp in the acute phase

Figure S2. Plasma NfL and GFAp concentrations at follow-up >175 days

**Supplementary tables……………………………………………………………………………………………………...**

Table S1. Neurological symptoms reported at baseline in COVID-19 patients

Table S2. Logistic regression models assessing the effect on time to follow up on persisting neurological symptoms

Table S3. Logistic regression models assessing the effects of plasma biomarker concentrations on neurological symptoms

**Authors**

^1,2^Nelly Kanberg*, M.D.; ^3,4^Joel Simrén*, M.D.; ^1,2^Arvid Eden M.D.; ^1,2^Lars-Magnus Andersson, M.D.; ^5^Staffan Nilsson, Ph.D.; ^3,6,7,8^Nicholas J. Ashton, Ph.D.;^9,10^ Pär-Daniel Sundvall, M.D.; ^11^Bengt Nellgård, M.D.; ^3,4^Prof Kaj Blennow, M.D.; ^3,4,12,13^Prof Henrik Zetterberg, M.D.; ^1,2^ Prof Magnus Gisslén, M.D.

* contributed equally

^1^ Department of Infectious Diseases, Institute of Biomedicine, Sahlgrenska Academy, University of

Gothenburg, Gothenburg, Sweden

^2^ Region Västra Götaland, Sahlgrenska University Hospital, Department of Infectious Diseases, Gothenburg, Sweden

^3^ Department of Psychiatry and Neurochemistry, Institute of Neuroscience & Physiology, the Sahlgrenska Academy at the University of Gothenburg, Mölndal, Sweden

^4^ Clinical Neurochemistry Laboratory, Sahlgrenska University Hospital, Mölndal, Sweden

^5^ Department of Laboratory Medicine, Institute of Biomedicine, Sahlgrenska Academy, University of Gothenburg, Gothenburg, Sweden

^6^Wallenberg Centre for Molecular and Translational Medicine, University of Gothenburg, Gothenburg, Sweden;

^7^King’s College London, Institute of Psychiatry, Psychology and Neuroscience, Maurice Wohl Institute Clinical Neuroscience Institute, London, UK;

^8^NIHR Biomedical Research Centre for Mental Health and Biomedical Research Unit for Dementia at South London and Maudsley NHS Foundation, London, UK

^9^ Research, Education, Development & Innovation, Primary Health Care, Region Västra Götaland, Sweden.

^10^ General Practice/Family Medicine, School of Public Health and Community Medicine, Institute of Medicine, Sahlgrenska Academy, University of Gothenburg, Gothenburg, Sweden.

^11^ Department of Anesthesiology and Intesive care, Institution of Clinical Sciences, Sahlgrenska Academy, University of Gothenburg, Gothenburg, Sweden

^12^ Department of Neurodegenerative Disease, UCL Institute of Neurology, London, United Kingdom

^13^ UK Dementia Research Institute at UCL, London, United Kingdom

*Corresponding author*

Nelly Kanberg, M.D.

Department of Infectious Diseases, Institute of Biomedicine,

Sahlgrenska Academy at University of Gothenburg,

Sahlgrenska University Hospital

Gothenburg, Sweden

Tel.: +46 31 343 43 28

Email: nelly.kanberg@gu.se

**Figure S1. Correlations between NfL, GDF-15 and GFAp in the acute phase**

In the acute phase (<21 days after symptom onset), NfL, GDF-15, and GFAp significantly correlated each other in whole sample (A-C). Abbreviations: NfL, neurofilament light; GFAp, glial fibrillary acidic protein; GDF-15, growth differentiation factor 15. Correlation coefficients are derived from Pearson correlations

**Figure S2. Plasma NfL and GFAp concentrations at follow-up >175 days**

No significant differences were found across groups >175 days after symptom onset. NfL: p=0.7193

GFAp: p=0,0777. Abbreviations: NfL, neurofilament light; GFAp, glial fibrillary acidic protein

|  | **Mild (n = 24) n(%)** | **Moderate (n = 28) n(%)** | **Severe (n = 48) n(%)** |
| --- | --- | --- | --- |
| **Any** | 21 (88) | 24 (86) | 35 (73) |
| **Headache** | 6 (25) | 15 (54) | 20 (42) |
| **Myalgia** | 13(54) | 21(75) | 20 (42) |
| **Cognitive change** | 1 (4) | 1 (4) | 5 (10) |
| **Hyposmia** | 12 (50) | 11 (39) | 15 (31) |
| **Dysgeusia** | 12 (50) | 15 (54) | 16 (33) |

**Table S1. Neurological symptoms reported at baseline in COVID-19 patients**

**Table S2.** Logistic regression models assessing the effect on time (months) to follow up on persisting neurological symptoms.

|  | **OR** | ***p*-value** | **95% CI** |
| --- | --- | --- | --- |
| **Fatigue** | 0∙928 | ∙471 | ∙757–1∙137 |
| **Brain fog** | 0∙922 | ∙463 | ∙743–1∙145 |
| **Cognitive change** | 1∙156 | ∙256 | ∙900–1∙486 |
| **Hyposmia** | 1∙006 | ∙984 | ∙562–1∙800 |
| **Dysguesia** | 1∙287 | ∙394 | ∙720–2∙302 |

Abbreviations: OR, Odds ratio; CI, confidence interval.

**Table S3. Logistic regression models assessing the effects of log_10_ plasma biomarker concentrations at acute phase on neurological symptoms at follow-up**

| **NfL** |  | **OR** | ***p*-value** | **95% CI** |
| --- | --- | --- | --- | --- |
|  | Brain fog | ∙630 | ∙452 | ∙189 –2∙10 |
|  | Fatigue | ∙195 | ∙034 | ∙043 – ∙881 |
|  | Cognition | 1∙88 | ∙335 | ∙520 –6∙79 |
|  | Hyposmia | ∙034 | ∙234 | ∙000 –6∙23 |
|  | Dysguesia | 1∙17 | ∙908 | ∙078 –17∙6 |
| **GFAp** | Fatigue | ∙435 | ∙293 | ∙92 –2∙05 |
|  | Brain fog | ∙294 | ∙152 | ∙055 –1∙57 |
|  | Cognition | 1∙71 | ∙535 | ∙313 –9∙39 |
|  | Hyposmia | ∙015 | ∙169 | ∙000 –6∙04 |
|  | Dysguesia | ∙311 | ∙542 | ∙007 –13∙2 |

Abbreviations: OR, odds ratio; CI, confidence interval
